# Direct and Indirect Influence of Non-Native Neighbours on Pollination and Fruit Production of a Native Plant

**DOI:** 10.1371/journal.pone.0128595

**Published:** 2015-06-25

**Authors:** Ana Montero-Castaño, Montserrat Vilà

**Affiliations:** Departamento de Ecología Integrativa, Estación Biológica de Doñana (EBD), Agencia Estatal Consejo Superior de Investigaciones Científicas (CSIC), Sevilla, Spain; Helmholtz Centre for Environmental Research—UFZ, GERMANY

## Abstract

**Background:**

Entomophilous non-native plants can directly affect the pollination and reproductive success of native plant species and also indirectly, by altering the composition and abundance of floral resources in the invaded community. Separating direct from indirect effects is critical for understanding the mechanisms underlying the impacts of non-native species on recipient communities.

**Objectives:**

Our aims are: (a) to explore both the direct effect of the non-native *Hedysarum coronarium* and its indirect effect, mediated by the alteration of floral diversity, on the pollinator visitation rate and fructification of the native *Leopoldia comosa* and (b) to distinguish whether the effects of the non-native species were due to its floral display or to its vegetative interactions.

**Methods:**

We conducted field observations within a flower removal experimental setup (i.e. non-native species present, absent and with its inflorescences removed) at the neighbourhood scale.

**Results:**

Our study illustrates the complexity of mechanisms involved in the impacts of non-native species on native species. Overall, *Hedysarum* increased pollinator visitation rates to *Leopoldia* target plants as a result of direct and indirect effects acting in the same direction. Due to its floral display, *Hedysarum* exerted a direct magnet effect attracting visits to native target plants, especially those made by the honeybee. Indirectly, *Hedysarum* also increased the visitation rate of native target plants. Due to the competition for resources mediated by its vegetative parts, it decreased floral diversity in the neighbourhoods, which was negatively related to the visitation rate to native target plants. *Hedysarum* overall also increased the fructification of *Leopoldia* target plants, even though such an increase was the result of other indirect effects compensating for the observed negative indirect effect mediated by the decrease of floral diversity.

## Introduction

Entomophilous non-native plants usually become well integrated into resident plant-pollinator communities, affecting the pollination and reproductive success of native plants [1,2]. However, in many cases we are unaware of the underlying ecological mechanisms of such effects [3], and whether they are directly caused by the non-natives or indirectly through the modification of resident species’ presence and abundance [4].

**Fig 1:**
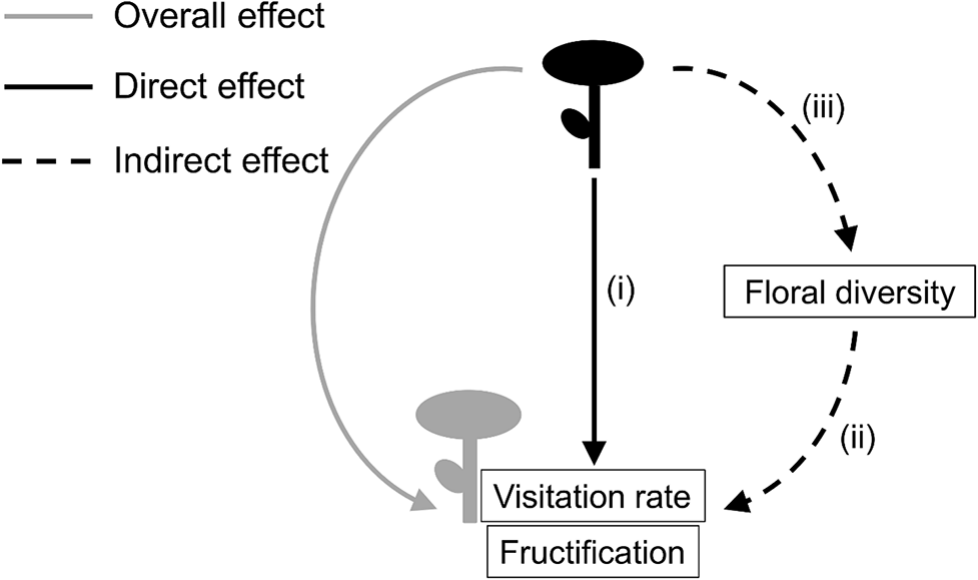
Schematic diagram on direct, indirect and overall effects. Effects of a non-native plant (black) on the visitation rate and fructification of a native target neighbour plant (grey). The overall effect (continuous grey arrow) can be the result of direct (continuous black arrow) and indirect (dashed black arrows) effects. The specific questions outlined in this study correspond to the numbered arrows.

Entomophilous non-native plants differ in their attractiveness to pollinators according to their abundance, spatial distribution, accessibility and/or quality of floral resources [5–7], and thus, they can directly (continuous black arrow in Fig 1) affect the pollination and subsequent reproductive success of native plants in the recipient community by altering the foraging behaviour of shared pollinators. For instance, native plant species growing in close proximity to highly attractive non-native plants may receive more visits than when growing alone, even when the total floral density does not differ [8], due to the magnet effect of non-native flowers [9]. Alternatively, highly attractive non-native plants may monopolize the visits of shared pollinators [10–12] or may increase heterospecific pollen deposition on native stigmas [13,14].

Furthermore, non-native plants can indirectly (dashed black arrow in Fig 1) affect the pollination and subsequent reproductive success of a particular native species by altering the composition, abundance and diversity of floral resources in the recipient community [5]. This can be due to their own floral offer or the result of competition for abiotic resources (i.e. nutrients, water and light), allelopathy or the interaction with shared herbivores or pathogens [15–17] associated with their vegetative parts (vegetative interactions, hereafter). These community changes in floral resources will influence the foraging behaviour of pollinators in response to the new floral environment [18,19] and according to their diet breadth, flower constancy, flying distance between consecutive visits, etc. [6,20]. For instance, pollinators might be attracted to a highly diverse patch if they have generalist diets or if they seek multiple resources (i.e. pollen, nectar, mates, hosting sites) provided by different plant species [21]. However, that might also imply a high heterospecific pollen deposition on native stigmas [22]. On the other hand, specialist pollinators or those showing high flower constancy may mainly respond to the abundance of their preferred resource in the neighbourhood, irrespectively to the floral diversity [19].

In sum, the overall effect (continuous grey arrow in Fig 1) of a non-native plant species on the pollination and reproduction of a particular native plant species depends on both direct and indirect effects, which can be mediated by the floral display of the non-native or by its vegetative interactions.

Pollinator interactions respond to the characteristics of the community at different spatial scales, including the neighbourhood scale (i.e. short adjacent areas) [23–26], whereas vegetative interactions (i.e. competition for soil resources and light) mainly occur at the neighbourhood scale [27–30]. Therefore, the neighbourhood is an ideal spatial scale at which to simultaneously explore the underlying mechanisms of both direct and indirect effects of non-native plants on plant-pollinator interactions at the individual plant level.

Here we present a study in which we explore the direct and indirect effects of the non-native and high rewarding *Hedysarum coronarium* on the pollination and reproductive success of the native *Leopoldia comosa*, at the neighbourhood scale. We compared native target plants growing in neighbourhoods where we manually removed the non-native inflorescences with those growing in both invaded and never invaded neighbourhoods. We outline the following specific questions (corresponding to each numbered arrow in Fig 1): (i) Does the non-native plant directly affect the visitation rate and fructification of the native plants, and is the effect mediated by its floral display or by vegetative interactions?; (ii) Does the floral diversity in the neighbourhood affect the visitation rate and fructification of the native plants?; (iii) Does the non-native plant alter the floral diversity in the neighbourhood, and is the effect mediated by its floral display or by vegetative interactions?

## Materials and Methods

### Study species and study site

The non-native study species was *Hedysarum coronarium* L. (hereafter *Hedysarum*), a short-lived N-fixing perennial legume [31] with either erect (0.8 m average height) or prostrate growth [32]. *Hedysarum* inflorescences are racemes with up to 30, approximately 1cm long, zygomorphic pink flowers rich in pollen and nectar that bloom during April and May. Its flowers, which are primarily pollinated by bees [33,34], are self-compatible but present high out-crossing rates [33,35]. In our study area, honeybees accounted for more than the 90% of the visits [36]. *Hedysarum* is native to the south-western Mediterranean Basin [37] but it has been introduced into other semiarid regions of the Mediterranean Basin as a forage plant as well as for erosion control, re-vegetation and high quality honey production [34,35,38]. Currently, whether native or introduced, it grows in many Mediterranean Basin countries, extending from Turkey to Spain [38].

In Menorca (Balearic Islands, Spain), where we conducted our study, *Hedysarum* was introduced between the end of the 18^th^ and the beginning of the 19^th^ centuries [39]. Since 1860 it has been used in a traditional cyclical agro-farming system [40]. *Hedysarum* subsequently escaped from cultivated fields and at present has become naturalized in natural and semi-natural areas such as ditches, old-fields, field edges and ruderal areas [41].

As the native study species we selected *Leopoldia comosa* Parl. (syn. *Muscari comosum* Mill.) (hereafter *Leopoldia*), a geophyte native to the Mediterranean Basin [42]. It is a 30 cm tall herb with prostrate leaves and a raceme inflorescence of up to 20 fertile greenish flowers with the floral pieces completely united in 2–3 mm wide actinomorphic cylinders. At the top of the inflorescence there is a group of sterile violet flowers [42]. *Leopoldia* was chosen as the target native species because it met the following requirements: (i) it grows in communities in which *Hedysarum* has become naturalized; (ii) its flowering phenology overlaps with that of *Hedysarum*; (iii) its reproduction is sexual [43] and, although it is self-compatible [44,45], it highly depends on out-crossing (S1 Table); (iv) it shares some pollinator species with *Hedysarum* [36].

The study site comprised a 3 ha shrubland (40°2.468’N, 4°5.845’E) dominated by *Olea europaea* ssp. *sylvestris* and *Pistacia lentiscus* with a rich herbaceous understory with up to 20 flowering plant species belonging to seven different families (S2 Table). *Hedysarum* has been present in the study site for at least ten years (*landowner’s communication*), which represents a sufficient duration for the study community to respond to the invasion in terms of species abundance and composition, as has been shown for other invaded herbaceous communities e.g. [46].

### Ethics statement

Both study species are common and non-protected in Menorca and their manipulation does not require any specific permission, particularly *Hedysarum*, as it is not native to the island [47]. The field work was carried out on private land with the knowledge and consent of the owner.

### Experimental design and neighbourhood characterization

In spring 2010 we selected 43 *Leopoldia* target plants, with a minimum distance of 2 m between individuals, and we established a 1 m radius neighbourhood around each target plant. The size of the neighbourhood, though smaller than in other pollination neighbourhood studies e.g. [25,26,47], was established on the basis of previous results regarding the area of influence of *Hedysarum* on the pollination of coexisting native plants (Montero-Castaño and Vilà, *submitted*). In this previous study, we conducted a total of 185 pollinator censuses of 15 min (i.e. 46.25 hours of observation) on all native co-flowering plant species within a 20 x 20 m^2^ invaded plot. We found that for the pool of native plants, including *Leopoldia*, visitation rates were three fold higher in individual plants with *Hedysarum* flowers ≤ 1 m away, than for those located > 1 m from *Hedysarum* flowers (1.15 ± 0.31 and 0.34 ± 0.13 visits/flower/hour, respectively; N = 185, Z = -3.677, P < 0.001; S1 Text). In addition, considering the height of the two species and the sometimes prostrate growth of *Hedysarum* [32], 1 m radius should be a suitable distance to detect vegetative interactions [48].

**Fig 2:**
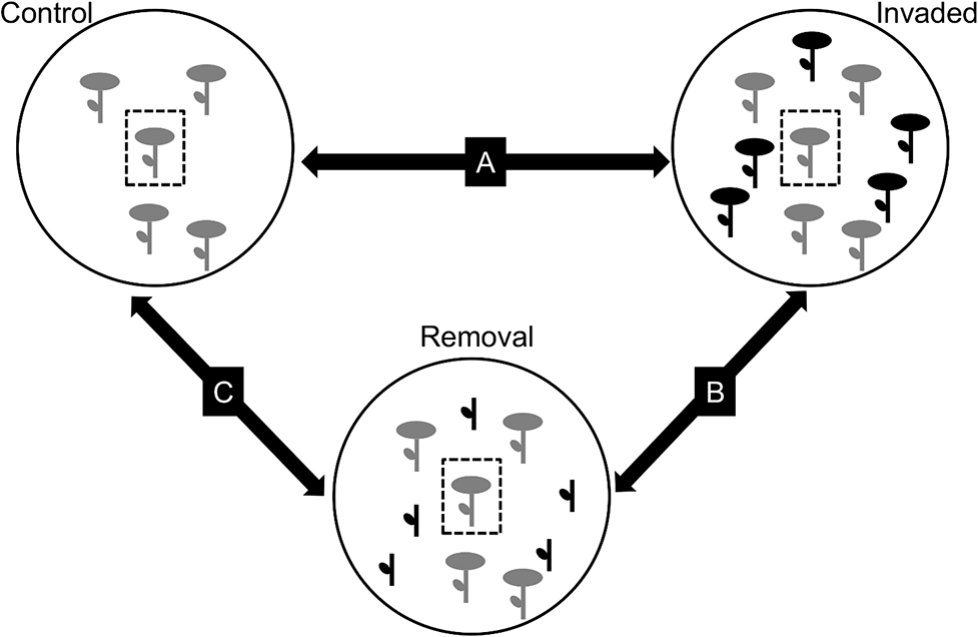
Neighbourhood treatments. The non-native *Hedysarum* is represented in black and the native species, whether *Leopoldia* or others, are represented in grey. Target *Leopoldia* plants are represented inside dashed squares. Arrows represent the different comparisons done to assess the overall effect of *Hedysarum* (A); the effects mediated by *Hedysarum* floral display (B); and the effects due to *Hedysarum* vegetative parts (vegetative interaction) (C).

Within the 1 m radius around *Leopoldia* target plants we randomly established three neighbourhood treatments according to the presence of *Hedysarum*: (i) Control, *Hedysarum* plants absent; (ii) Invaded, *Hedysarum* flowering plants present; and (iii) Removal, *Hedysarum* plants with clipped inflorescences but intact vegetative parts present (Fig 2). Overall, there were 14 *Leopoldia* target plants without non-native neighbours (Control); 11 *Leopoldia* target plants with *Hedysarum* individuals in their neighbourhoods (Invaded) and 18 *Leopoldia* target plants with manually clipped *Hedysarum* inflorescences in their neighbourhood (Removal). Clipping was conducted as often as it was necessary, usually every 3–4 days, in order to ensure that no new inflorescences were able to bloom during the sampling season. *Hedysarum* cover did not differ between Invaded and Removal treatments (N = 29, t = -0.171, P-value = 0.866). The presence and abundance of the other floral resources in the neighbourhood, including non-target *Leopoldia* individuals, were not manipulated in any of the treatments.

These three treatments allowed us to explore the overall effect of *Hedysarum* on the pollination and fructification of *Leopoldia* (Control *vs*. Invaded; arrow A in Fig 2), and to isolate the effect mediated by the floral display of *Hedysarum* (Invaded *vs*. Removal; arrow B in Fig 2) from the effect associated with the vegetative parts of *Hedysarum* (vegetative interactions) (Control *vs*. Removal; arrow C in Fig 2).

In each neighbourhood we established eight 0.4 x 0.4 m^2^ quadrants, two located at each of the four cardinal directions. In each quadrant we counted all open flowers for all flowering species, excluding the target plants. We then extrapolated these values to the total area of the neighbourhood. Main neighbourhood characteristics can be found in Table 1. We estimated the true floral diversity (^*q*^ *D*) [49], which is equivalent to the natural exponent of the Shannon index (*H’*) calculated with the natural algorithm [50]. ^*q*^ *D* is calculated as the inverse of the geometric mean species proportional abundance; i.e., when *q* = 1 and, therefore, each species is weighted by its proportional abundance [51]:

Dq=exp(−∑i=1Rpilnpi)=exp(H')

where *p*_*i*_ is the proportion of flowers of the species *i*, *R* is the number of flowering plant species and *ln* is the natural logarithm.

**Table 1:** Ranges (min and max values) of main characteristics of 1 m radius neighbourhoods around *Leopoldia* target plants according to the presence of non-native *Hedysarum* plants: (i) Control, *Hedysarum* plants absent; (ii) Invaded, *Hedysarum* flowering plants present; and (iii) Removal, *Hedysarum* plants with clipped inflorescences but intact vegetative parts present.

Treatment	Target plant flowers*	*Hedysarum* cover (%)	*Hedysarum* flowers	*Leopoldia* flowers	Total flowers	Flowering species	Floral diversity (^*1*^ *D*)	Closest *Hedysarum* flower from target plant (m)
Control	6–26	-	-	0–215.6	12.3–215.6	1–6	0–4.57	-
Invaded	8–24	15.6–65.6	4.9–102.9	0–245.0	24.5–279.3	1–3	0–2.83	0.1–1
Removal	4–22	3.1–81.3	-	0–58.8	0–78.4	0–5	0–2.80	-

* Total number of flowers observed during the study

### Pollination censuses and fruit production

Pollination censuses were conducted on each of the 43 *Leopoldia* target plants between the 10^th^ and the 24^th^ of April during sunny, warm (≥ 17°C) and non-windy days, from 10 a.m. to 6 p.m. Each census lasted 15 min during which we noted the number of visits of each pollinator species. A visitor was considered a pollinator if it entered a flower and touched the sexual parts of the plant. After each observation period we counted and marked with a permanent pen the peduncle of all open flowers of the target plant [52]. As the flowers of this species do not last more than one day (Montero-Castaño, *personal observation*), estimates derived from our censuses are highly accurate. Each *Leopoldia* target plant was observed three times, with the exception of three target plants that could only be observed twice because their bloom finished before the end of the field season. Observations of a single target plant were conducted on different days, and were randomly distributed throughout the day. In total we conducted 147 censuses (36.75 h). For each target plant, we estimated the visitation rate (i.e. visits/flower/hour) as one of the response variables.

Similarity between pollinator communities among the neighbourhood treatments was tested using the proportional similarity index (*PS*; [53]), which takes into account not only the identity, but also the relative abundance of each pollinator species. This index is calculated as:

$$PS=\sum_{i=1}^{n}\min\left(p_{ia},p_{ib}\right)$$

where for *n* species *p*_*ia*_ is the relative abundance of pollinator species *i* at neighbourhood treatment *a* and *p*_*ib*_ is the relative abundance of pollinator species *i* at neighbourhood *b* (i.e. Invaded, Removal or Control). *PS* values range from 0 (no overlap between species composition) to 1 (complete overlap).

Approximately one month after the pollination censuses, we collected ripe fruits from a total of 569 marked flowers. Only ripe fruits from marked flowers (i.e., those observed during the censuses) were collected in order to more accurately link the reproductive success of target plants with the data obtained in the pollination censuses. The proportion of flowers that set fruit (hereafter, fructification) was also tested as a response variable.

### Statistical analyses

#### Do *Hedysarum* and the floral diversity in the neighbourhood affect the pollination and reproductive success of *Leopoldia*?

We analyzed the direct effect of *Hedysarum* on the visitation rate and fructification of *Leopoldia* target plants (arrow *i* in Fig 1), together with the influence of floral diversity in the neighbourhood (arrow *ii* in Fig 1), by building a generalized linear model for each response variable with treatment (Control, Invaded and Removal) and floral diversity as our explicative variables while controlling for the total flower density.

When analyzing the response variable visitation rate, the number of visits was kept as dependent variable and the logarithm of the number of observed flowers of *Leopoldia* target individuals and the logarithm of the hours of observation were included as offsets in the model [54]. To deal with overdispersion, the error distribution family was quasi-Poisson and “log” the link function. The same analysis was repeated excluding the visits made by the honeybee as this pollinator species accounted for more than 90% of pollinator visits to *Hedysarum*.

For fructification, error distribution family was the binomial and “logit” the link function.

For each response variable we conducted likelihood ratio tests (LRT) between the above described models and those missing the explanatory variable of interest (neighbourhood treatment or floral diversity) in order to calculate the significance of the coefficients estimated for each explanatory variable.

#### Does *Hedysarum* alter the floral diversity in the neighbourhood?

To analyze the indirect effect of *Hedysarum* on *Leopoldia* through the alteration of floral diversity in the neighbourhoods (arrow *iii* in Fig 1), we built a generalized linear model with treatment (Control, Invaded and Removal) as the fixed factor, floral diversity as the response variable and Gamma as the error distribution family (in order to deal with continuous and non-normal data) with the “log” link function.

All analyses were conducted in R [55]. *Post hoc* multilevel comparisons were conducted with the library *multcomp* [56]. Differences among the three neighbourhood treatments were tested based on the 95% confidence intervals of the estimates obtained for the *post hoc* multilevel comparisons. Data are available in S4 Table.

## Results

Overall, we observed nine insect species visiting *Leopoldia* plants, including six bees and three beetles; not all of them were observed in the three treatments (Table 2). The beetle *Psilothrix viridicoerulea* and the honeybee accounted for more than 90% of the visits to *Leopoldia* target plants in the Control and Invaded treatments. Meanwhile, in the Removal treatment the honeybee was never observed and *P*. *viridicoerulea* alone accounted for more than 80% of the visits. These two pollinator species, together with the bee *Chalicodoma sicula*, were shared between *Hedysarum* and *Leopoldia* (S3 Table). The proportional similarity index for pollinator species visiting *Leopoldia* plants was 0.82 between Control and Invaded, 0.79 between Control and Removal and 0.69 between Invaded and Removal treatments.

**Table 2:** Pollinator species and visits (%) to native *Leopoldia* plants in the study area during 147 censuses (36.75 h).

Species	Family	Order	Visits (%)		
			Control	Invaded	Removal
*Dasytes virens*	Melyridae	Coleoptera	0 (0.00)	0 (0.00)	2 (3.28)
*Oedemera sp*.	Cucujidae	Coleoptera	0 (0.00)	2 (3.03)	0 (0.00)
***Psilothrix viridicoerulea***	Melyridae	Coleoptera	20 (60.61)	26 (39.39)	49 (80.33)
***Apis mellifera***	Apidae	Hymenoptera	10 (30.30)	37 (56.06)	0 (0.00)
***Chalicodoma sicula***	Megachilidae	Hymenoptera	2 (6.06)	0 (0.00)	0 (0.00)
*Lasioglossum sp*.	Halictidae	Hymenoptera	1 (3.03)	0 (0.00)	0 (0.00)
*Osmia niveata*	Megachilidae	Hymenoptera	0 (0.00)	0 (0.00)	3 (4.92)
*Plagiolephis pygmaea*	Formicidae	Hymenoptera	0 (0.00)	1 (1.52)	5 (8.20)
*Platygastridae sp*.	Platygastridae	Hymenoptera	0 (0.00)	0 (0.00)	2 (3.28)

Species in bold letters are the ones shared with non-native *Hedysarum* plants (see S3 Table). Total number and percentage (in brackets) of visits achieved by each pollinator species in each neighbourhood treatment are also given.

### Do *Hedysarum* and the floral diversity in the neighbourhood affect the pollination and reproductive success of *Leopoldia*?

**Fig 3:**
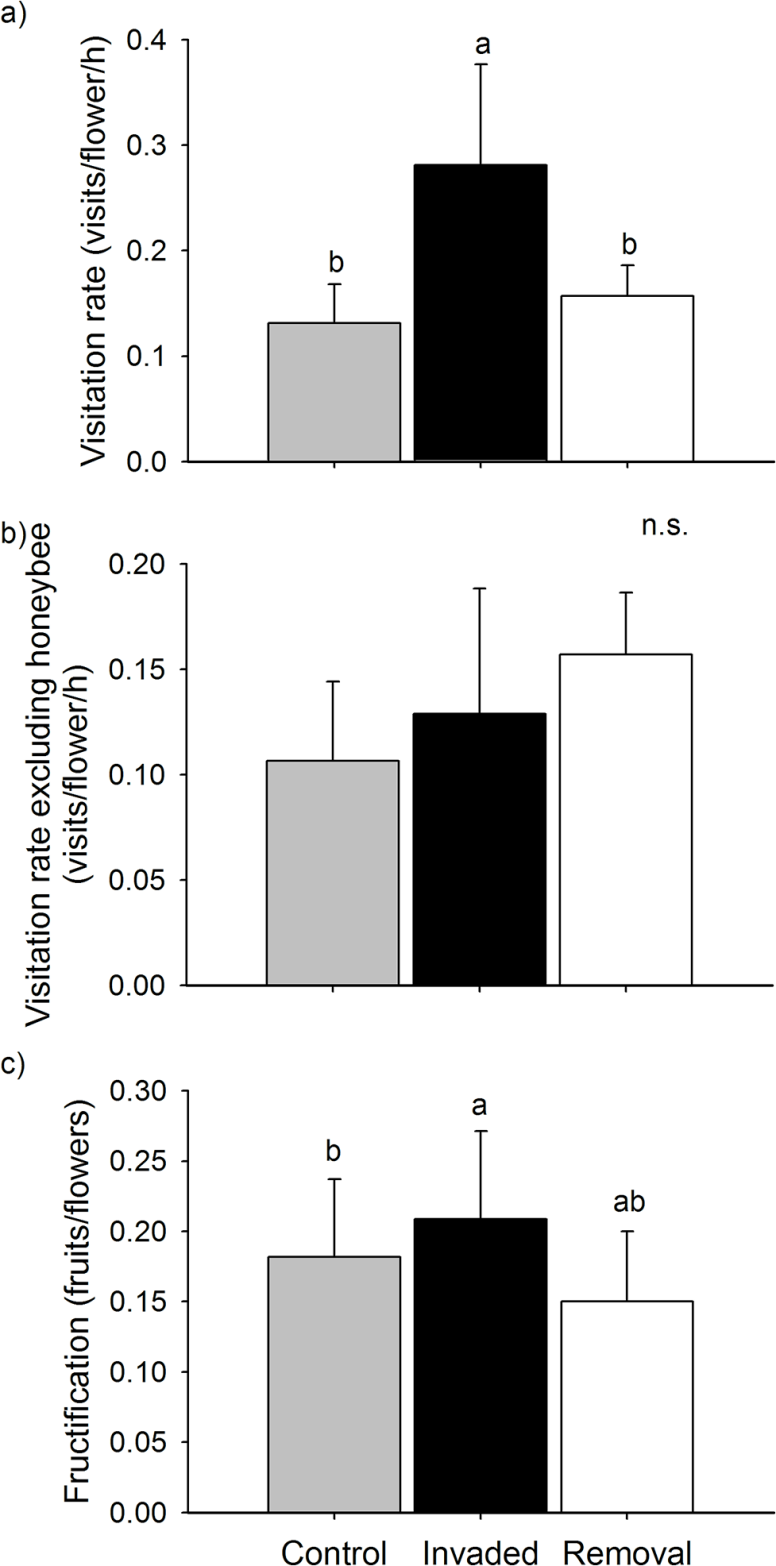
Effect of *Hedysarum* and the floral diversity on the pollination and reproductive success of *Leopoldia*. Mean + SE (a) total pollinator visitation rate (i.e. visits/flower/hour), (b) pollinator visitation rate excluding the honeybee and (c) fructification (i.e. proportion of observed flowers that set fruit) in *Leopoldia* target plants in Control (grey bar), Invaded (black) and Removal (bold) neighbourhood treatments. Significant differences are represented by different letters above bars according to the 95% confidence intervals of the estimates obtained for the *post hoc* multilevel comparisons conducted for the models, which included the floral diversity and the density of flowers as covariates.

Due to a direct effect mediated by its floral display, *Hedysarum* contributed to the overall increase of the pollinator visitation rate to *Leopoldia* target plants. The visitation rate to *Leopoldia* target plants differed among treatments (LTR Chisq = -22.911, P-value = 0.017) and decreased in conjunction with the floral diversity in their neighbourhoods (LRT Chisq = -13.066, P-value = 0.031). The visitation rate to *Leopoldia* in the Invaded treatment was higher than in the Control and Removal treatments, while no differences were found between Control and Removal treatments (Table 3, Fig 3A).

**Table 3:** *Post hoc* multiple comparisons among neighbourhood treatments for the response variables visitation rate (total, and excluding the honeybee), fructification and floral diversity in the neighbourhood of *Leopoldia* target plants.

Response variable	Neighbourhood comparison	Estimate	SE	95% CI bounds	
				Lower	Upper
Visitation rate	Control *vs*. Invaded	-0.736	0.363	-1.447	-0.025^*^
	Removal *vs*. Invaded	-0.874	0.332	-1.525	-0.223^*^
	Removal *vs*. Control	-0.138	0.418	-0.957	0.682
Visitation rate excluding the honeybee	Control *vs*. Invaded	-0.309	0.451	-1.192	0.574
	Removal *vs*. Invaded	-0.464	0.397	-1.242	0.315
	Removal *vs*. Control	-0.155	0.429	-0.996	0.686
Fructification	Control *vs*. Invaded	-0.650	0.322	-1.281	-0.018^*^
	Removal *vs*. Invaded	-0.554	0.301	-1.145	0.036
	Removal *vs*. Control	0.095	0.305	-0.502	0.692
Floral Diversity (^*1*^ *D*)	Control *vs*. Invaded	0.329	0.172	-0.008	0.667^.^
	Removal *vs*. Invaded	-0.031	0.164	-0.351	0.290
	Removal *vs*. Control	-0.360	0.152	-0.658	-0.062^*^

Confidence intervals of the estimates not crossing zero mean significant differences and are marked with an asterisk. Marginally significant differences are marked with a dot.

When excluding the visits of honeybees from the analysis, *Hedysarum* did not affect the visitation rate to *Leopoldia* target plants (Table 3, Fig 3B). The visitation rate to *Leopoldia* target plants did not differ among treatments (LRT Chisq = -3.226, P-value = 0.517), nor was it altered by the floral diversity (LRT Chisq = -4.315, P-value = 0.184).

Overall, *Hedysarum* increased the fructification of *Leopoldia* target plants. The fructification of *Leopoldia* target plants differed slightly among treatments (LTR Chisq = -4.832, P-value = 0.089) and increased together with floral diversity in their neighbourhoods (LRT Chisq = -3.923, P-value = 0.048). The fructification of *Leopoldia* in the Invaded treatment was slightly higher than in the Control treatment but did not differ from that in the Removal treatment. Fructification did not differ between the Control and the Removal treatments (Table 3, Fig 3C).

### Does *Hedysarum* alter the floral diversity in the neighbourhood?

**Fig 4:**
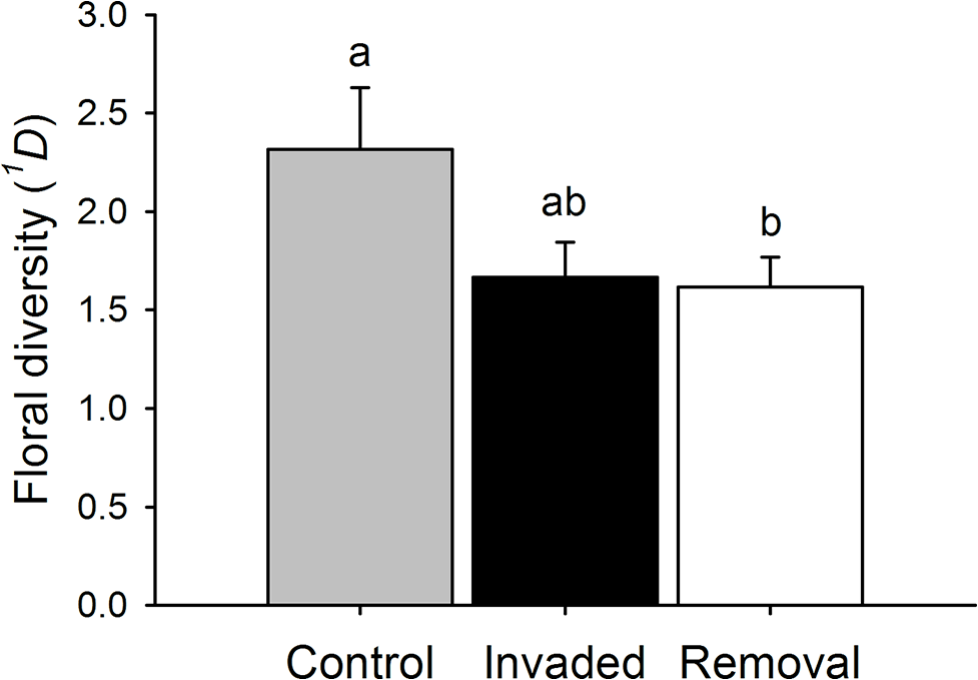
Effect of *Hedysarum* on the floral diversity. Mean + SE true floral diversity (^*1*^ *D*) in Control (grey bar), Invaded (black) and Removal (bold) neighbourhood treatments. Significant differences are represented by different letters above bars according to the 95% confidence intervals of the estimates obtained for the *post hoc* multilevel comparisons conducted for the model, which included the density of flowers as covariate.

*Hedysarum* decreased the floral diversity in its neighbourhood due to vegetative interactions. The floral diversity in *Leopoldia* neighbourhoods differed among treatments (LTR Chisq = 7.105, P-value = 0.038). It was higher in the Control treatment than in the Invaded and Removal treatments, though with the Invaded the difference was only marginally significant. Meanwhile, the floral diversity in the Invaded treatment did not differ between the Invaded and Removal treatments (Table 3, Fig 4).

## Discussion

**Fig 5:**
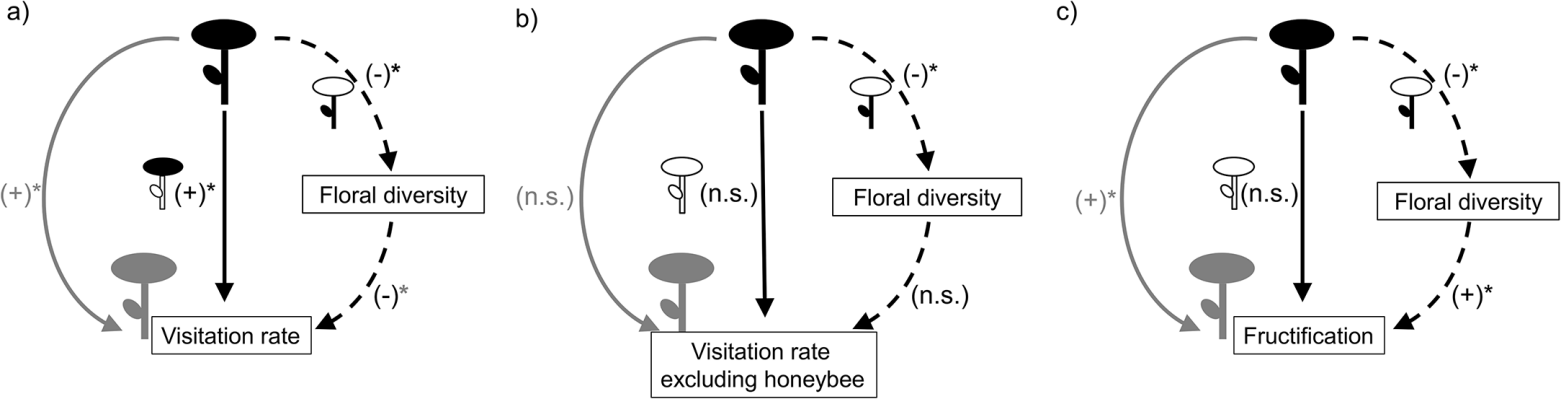
Schematic diagram resuming the results. (a) Total visitation rate, (b) visitation rate excluding the honeybee and (c) fructification in *Leopoldia* target plants. The non-native *Hedysarum* is represented in black while the native *Leopoldia* is represented in grey. Grey continuous arrows represent the overall effect of *Hedysarum* on *Leopoldia* target plants while black continuous and dashed arrows represent direct and indirect effects, respectively. The sign of the effect is given in brackets next to each arrow. Whether the effect is mediated by the vegetative parts (vegetative interaction) or by the floral display of *Hedysarum*, is indicated by coloring the part involved in the effect and leaving the not involved in bold.

We found that the non-native *Hedysarum* affected the pollination and reproductive success of its native neighbours both directly and indirectly, the latter specifically by decreasing the floral diversity in its neighbourhood. Both the magnitude and direction of such effects vary across the different stages of the pollination process of *Leopoldia* (i.e., from the visits it receives to the fruits it produces). In addition, our experimental approach has allowed us to disentangle whether these direct and indirect effects are mediated by the floral display or by vegetative interactions associated with the vegetative parts of the non-native *Hedysarum* (Fig 5).

### Direct effects

Owing to its floral display, *Hedysarum* exerted a magnet effect [8,57] on *Leopoldia* neighbouring plants by attracting the visits of shared pollinators (Fig 5A). *Leopoldia*, which usually grows intermingled in the herb vegetation layer (Montero-Castaño, *personal observation*), might benefit greatly from the presence of attractive and generalist plant species like *Hedysarum*.

The honeybee was the main pollinator that responded to the magnet effect, accounting for most *Leopoldia* visits when *Hedysarum* was present in its neighbourhood. This pollinator species has a generalized diet [58] and shows an intensive foraging behaviour with a short flying distance between two consecutive visits [59]. It might be beneficial for the honeybee to make consecutive interspecific visits, as long as flowers are within short flying distances. Besides, the honeybee may respond to many other magnet effects between plant species with contrasting attractiveness to pollinators as it is an abundant and ubiquitous species [60].

However, the magnet effect did not translate into a higher reproductive success of *Leopoldia* target plants in terms of the proportion of flowers setting fruit (Fig 5C). That is, we found no evidence of a direct effect of *Hedysarum* on the reproductive success of *Leopoldia*. The decoupling of visitation rates and fructification is a common phenomenon which is both species and context dependent e.g. [61–63]. Fructification depends on the quantity and quality of the pollen available for the fertilization of ovules and on the resources available for fruit and seed production [64]. Magnet effects can compromise the quantity and quality of pollen deposition in several ways. First, interspecific visits between high attractive and low attractive plant species can imply heterospecific pollen deposition and conspecific pollen loss on target plants [65]. Second, if the attracted pollinators make several visits to the same individual plant, like the honeybee in our study system, geitonogamous pollen deposition may occur, thus decreasing the quality of pollen loads on target plants [66].

### Indirect effects

We also found evidence of indirect effects of *Hedysarum* on the pollination and reproductive success of *Leopoldia* target individuals mediated by the decrease of floral diversity in the neighbourhoods. The decrease in floral diversity was mediated by the vegetative parts of *Hedysarum* plants. Although we did not explore the mechanisms involved in such a decrease, competition for abiotic resources may occur. Non-native plants that are able to persist and invade a community usually outcompete natives for the use of soil resources and light [48]. As a result, the diversity of species, and subsequently the floral diversity in the recipient community, might decrease in invaded communities [67].

Floral diversity decreased the pollination visitation rate to *Leopoldia* target plants. Therefore, the indirect effect of *Hedysarum* on the *Leopoldia* visitation rate mediated by the alteration of floral diversity was positive (Fig 5A). In diverse floral neighbourhoods, flower richness and/or evenness are high. If the abundance of pollinators does not increase in conjunction with floral diversity [21,68] competition for pollinators among plant species may intensify and would be strong for low attractive plant species like *Leopoldia*. Even if the abundance of pollinators in neighbourhoods increases with floral diversity, as has been observed at different scales [19,69,70] such a competitive disadvantage might persist.

*Leopoldia* fructification was positively related to floral diversity. Therefore, the indirect effect of *Hedysarum* on *Leopoldia* fructification mediated by the alteration of floral diversity was negative (Fig 5C). In this case, the decoupling of visitation rates and fructification might be more related to the availability of resources for fruit and seed production [64]. In highly diverse neighbourhoods, coexisting plant species can avoid competition for resources for fruit and seed production due to resource partitioning or temporal segregation of their reproductive periods. Even more, the fructification of coexisting plant species in highly diverse neighbourhoods can be enhanced by facilitation or resource supply [71–73].

### Overall effects and conclusions

Overall, *Hedysarum* increased the visitation rate to *Leopoldia* target plants as a result of direct and indirect effects acting in the same direction. The honeybee played an important role in this pattern, as when the visits made by this pollinator were excluded from analyses, direct and indirect effects disappeared.

The reproductive success of *Leopoldia* target plants also increased with the presence of *Hedysarum* growing in the neighbourhood, even though it was not the result of both direct and indirect effects acting in the same direction. For this response variable, we found no evidence of a direct effect of *Hedysarum*. Meanwhile, other indirect effects [52] should be acting and compensating for the negative indirect effect mediated by the decrease of floral diversity. For instance, as *Hedysarum* is a legume species, its indirect effect on natives’ fructification mediated by the alteration of soil nitrogen availability [71] deserves future exploration. Therefore, in order to understand the impacts of non-native plants on the pollination of native plants both direct and indirect effects, which are also highly context-dependent, must be considered.

Despite the observed quick response of pollinators to our manipulative experiment, long term effects could be different due to lag-times in pollinator responses [74,75]. For instance, we could expect pollinator communities in Removal treatments to be more similar to those in Control treatments in the long-term. However, in herbaceous communities like the one studied here, such long term effects would be confounded by the natural plant species turnover.

In addition, our experimental approach has allowed us to assess the underlying mechanisms of the effect of non-native species on native target plants; specifically, whether the effect of non-native plants is mediated by vegetative interactions (i.e. competition for space, light, nutrients, water), or whether such an effect is mediated by their floral display (i.e. through shared pollinators).

## Supporting Information

S1 Table Reproductive biology of the native study species. Differences in fruit production in *Leopoldia* non manipulated flowers (control) and in flowers bagged with a tea bag to avoid any pollen transfer mediated by insects (autogamy). Each treatment was randomly assigned to one out of two flowers in 30 individuals selected in the study site in spring 2009 (though afterwards some flowers were damaged or missing when and could not be included in the analysis). Differences in fruit production between treatments were analysed with a Chi-square test.
(XLSX)

S2 Table Native flowering plant community. Flowering plant species and family found in the pool of the 43 study neighbourhoods, including the two study species *Hedysarum coronarium* and *Leopoldia comosa*, highlighted in bold letters.
(XLSX)

S3 Table Pollinator community of the non-native study species. Pollinator species of *Hedysarum* observed during 138 censuses (34.5 h). Censuses were carried out on *Hedysarum* individuals distributed among four 20 X 20 m^2^ independent plots were *Hedysarum* was naturalized *sensu* [sensu 1] in Menorca (Site 1: 39° 56.395'N, 4° 15.052'E; Site 2: 39° 56.677'N, 4° 14.892'E; Site 3: 39° 56.191'N, 4° 13.000'E; Site 4: 40° 2.429'N, 4° 5.908'E). Percentages of total number of visits achieved for each pollinator species are also given.
(XLSX)

S4 Table Database. Data underlying the findings of this study.
(XLSX)

S1 Text Justification of neighbourhood size. (DOCX)
