# Supplementary material for: Direct and Indirect Influence of Non-Native Neighbours on Pollination and Fruit Production of a Native Plant
Source: PLoS One. 2015 Jun 25;10(6):e0128595. doi: 10.1371/journal.pone.0128595 (PMC4482463; doi:10.1371/journal.pone.0128595)
Supplement: S1 Text — (DOCX) [file pone.0128595.s005.docx]

**Supporting Information**

**Direct and indirect influence of non-native neighbours on pollination and fruit production of a native plant**

**Ana Montero-Castaño^1*^ and** **Montserrat Vilà^1^**

^1^ Departamento de Ecología Integrativa, Estación Biológica de Doñana (EBD), Agencia Estatal Consejo Superior de Investigaciones Científicas (CSIC), Sevilla, Spain.

^*^Corresponding author:

E-mail: [anamontero@ebd.csic.es](mailto:anamontero@ebd.csic.es) (AMC)

As part of a complementary study, we conducted a total of 185 censuses on the entire flowering plant community in a 20x20 m^2^ plot located within the study site and following the same protocol than in this study (Montero-Castaño and Vilà, *unpublished*).

We found that the visitation rates observed in those individuals whose closest *Hedysarum* flower was <1m apart, were more than threefold higher than in individuals whose closest *Hedysarum* flowers were more distant (1.15 ± 0.31 and 0.34 ± 0.13 visits/flower/hour, respectively; Fig. S2.1), when considering the whole pool of native co-flowering plants.

We statistically analyzed such differences in visitation rates by building a generalized linear model with distance to the closest *Hedysarum* flower (≤ 1 m *vs.* > 1m) as fixed factor and plant species as random factor. The logarithm of the number of observed flowers was included as an offset and the error distribution family was quasi-Poisson to deal with overdispersion. The analysis was conducted with the library *lme4* of the R statistical computing environment [2].

We found differences to be statistically significant (N = 185, Z = -3.677, P < 0.001).

**
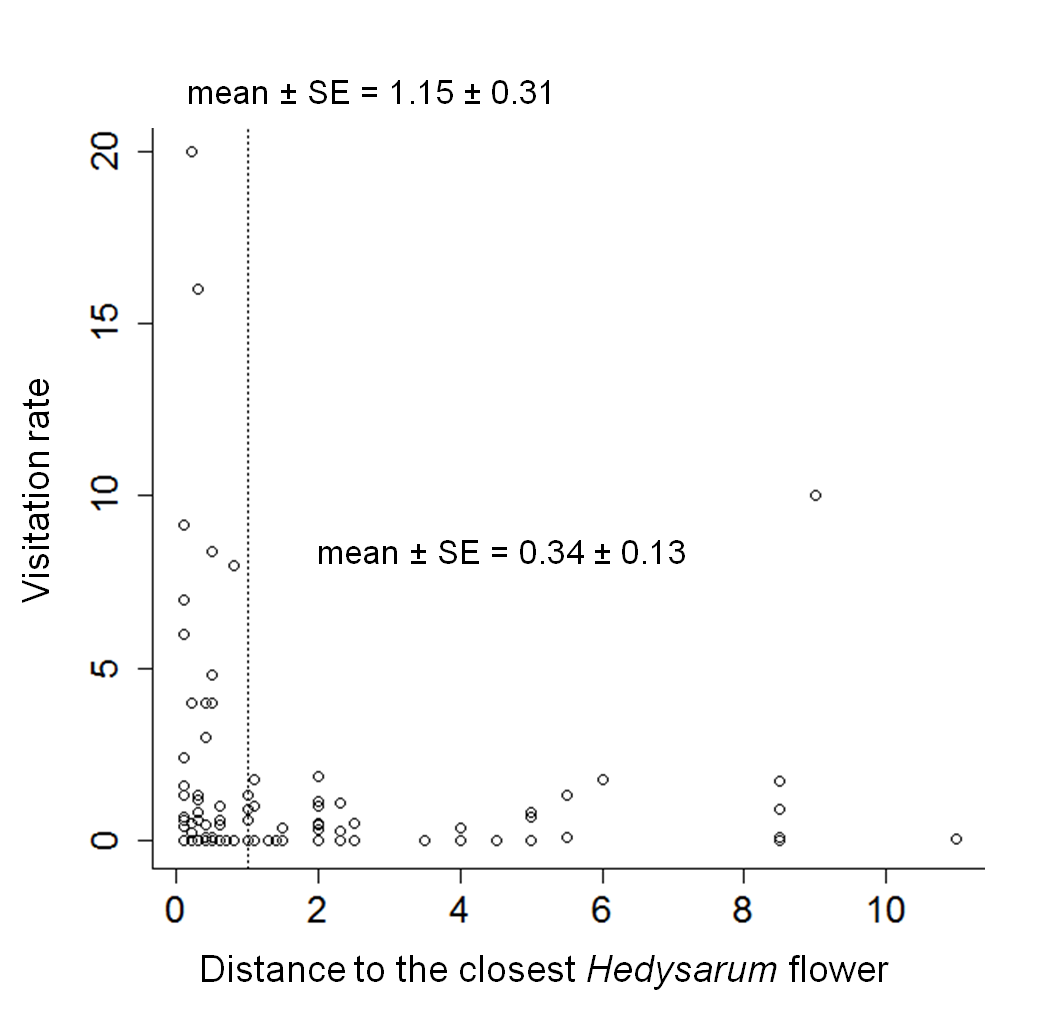
**

**S4 Fig. Justification of neighbourhood size.** Relationship between visitation rate (visits/flower/hour) and the distance to the closest *Hedysarum* flower for the pool of co-flowering native plants in the study site. On the left of the dashed line, values for distances <1m and on the right, values for distances >1m. Mean ± SE of visitation rate for both cases are given.

**References**

1. Pyšek P, Richardson DM, Rejmanek M, Webster GL, Williamson M, Kirschner, J. Alien plants in checklists and floras: towards better communication between taxonomists and ecologists. Taxon 2004; 53: 131–143.

2. R Development Core Team. R: A language and environment for statistical computing. 2001. Available: http://www.r-project.org/.
